# Supplementary material for: Biomarkers of dairy fat intake, incident cardiovascular disease, and all-cause mortality: A cohort study, systematic review, and meta-analysis
Source: PLoS Med. 2021 Sep 21;18(9):e1003763. doi: 10.1371/journal.pmed.1003763 (PMC8454979; doi:10.1371/journal.pmed.1003763)
Supplement: S1 Protocol — (DOCX) [file pmed.1003763.s003.docx]

**S1 Protocol. Prespecified analytical plan for 60YO cohort study**

***Analytical plan***

**Association of serum biomarkers of dairy fat intake with incident CVD and all-cause mortality**

**Hypotheses:** We hypothesize that serum 15:0 will be associated with lower incident CVD and all-cause mortality.

**Specific** **Aim 1:** To investigate whether circulating 15:0 is associated with incident CVD and all-cause mortality in the Stockholm Cohort of 60-Year-Olds (60YO).

**Specific Aim 2:** To investigate potential effect modification by sex, long-chain n-3 PUFA biomarker concentration (i.e., sum of EPA and DHA), and BMI in the association of circulating 15:0 and 17:0 with incident CVD and all-cause mortality.

**Methods:**

Study population

All participants with measured 15:0 in serum cholesteryl esters (CE) will be included in the analysis.

Exposure

The exposures (15:0 in CE) will be analyzed as a continuous variable (% total fatty acids, per 1 interquintile range increment) and in quartiles. Nonlinear relationships will be tested using restricted cubic splines.

Outcomes

1. Incident CVD (defined as the composite of non-fatal MI, CHD death, sudden cardiac death, or fatal ischemic stroke).
2. All-cause mortality.

Statistical model

Cox proportional hazards models, with robust variance, will be used to estimate the hazard ratio for all-cause and CVD mortality. Follow-up time will be calculated from baseline (biomarker measurement) to date of failure, end of follow-up, loss to follow-up, or death, whichever occurred first. Laplace regression will be used to estimate percentile differences in time to first CVD event or death. Three models will be used:

1. Crude.
2. Age- and sex-adjusted.
3. Mulitvariable adjusted (including the following covariates: age, sex, BMI, education, smoking, physical activity, alcohol intake, diabetes status, treated hypertension, treated hypercholesterolemia, history of CVD [only for analysis of all-cause mortality] and sum of EPA and DHA biomarker concentrations).

Multiple imputation will be used for missing covariates. To evaluate effect modification, stratified analyses will be conducted for the following variables: sex (males, females); sum of EPA and DHA biomarker concentrations (< or ≥ median value); and BMI (<25 kg/m^2^; ≥ 25 kg/m^2^).

Sensitivity analyses

Participants will be censored at the first 10 years of follow-up to minimize exposure misclassification due to within-person variation over time. We will exclude cases in the first two years of follow-up to avoid reverse causation.
