# Supplementary material for: Biomarkers of dairy fat intake, incident cardiovascular disease, and all-cause mortality: A cohort study, systematic review, and meta-analysis
Source: PLoS Med. 2021 Sep 21;18(9):e1003763. doi: 10.1371/journal.pmed.1003763 (PMC8454979; doi:10.1371/journal.pmed.1003763)
Supplement: S1 Text — (DOCX) [file pmed.1003763.s004.docx]

**S1 Text. Health screening and questionnaire**

Participants underwent a health screening, including blood sampling and completion of an extensive questionnaire on disease history, health status, medication, and lifestyle (e.g., dietary habits, physical activity, alcohol intake, and smoking). All study participants underwent physical examinations in which anthropometric measures and blood pressure were recorded. Blood samples were collected after overnight fasting and serum was stored at −80°C until analysis. Enzymatic methods (Bayer Diagnostics, Tarrytown, NY) were used to measure serum cholesterol and triglycerides; and serum high-density lipoprotein cholesterol was analyzed enzymatically, after isolation of low- and very-low-density lipoprotein cholesterol (Boehringer Mannheim GmbH, Germany).

The questionnaire collected information on alcohol intake (categorised as <1 drink (14 g ethanol)/week, <1 drink/day, <2 drinks/day and ≥ 2 drinks/day); education level (primary school, secondary school and university education); physical activity in leisure time (sedentary, light intensity, regular moderate intensity and regular high intensity); smoking (never, former and current smoker); diabetes mellitus (self-reported diabetes diagnosis, medication-treated diabetes mellitus, or fasting serum glucose ≥7 mmol/L); drug-treated hypertension and hypercholesterolemia (self-reported use of drugs). The questionnaire also included 17 questions regarding dietary quality with special emphasis on dietary fat intake.[1] We calculated a dairy score as previously described,[1] and categorized consumption of vegetables (≥1 time/day, and <1 time/day), fruits and berries (≥1 time/day, and <1 time/day), lean fish (≥1 time/week, and <1 time/week), oily fish (≥1 time/week, and <1 time/week), and processed meat as main dish (≥1 time/week, and <1 time/week).

*Supplementary Reference*

1. Laguzzi F, Alsharari Z, Risérus U, Vikström M, Sjögren P, Gigante B, et al. Cross-sectional relationships between dietary fat intake and serum cholesterol fatty acids in a Swedish cohort of 60-year-old men and women. J Hum Nutr Diet,. 2016;29(3):325-37. doi: 10.1111/jhn.12336.
