# Supplementary material for: Biomarkers of dairy fat intake, incident cardiovascular disease, and all-cause mortality: A cohort study, systematic review, and meta-analysis
Source: PLoS Med. 2021 Sep 21;18(9):e1003763. doi: 10.1371/journal.pmed.1003763 (PMC8454979; doi:10.1371/journal.pmed.1003763)
Supplement: S2 Text — (DOCX) [file pmed.1003763.s005.docx]

**S2 Text. Search strategy and methods**

*Search strategy*

**(“pentadecanoic acid” OR “heptadecanoic acid” OR “trans-palmitoleic acid” OR “fatty acid*” OR “milk fat” OR “dairy fat” OR “odd chain fatty acid”) OR (“Fatty Acids” [Mesh]) AND (“blood” OR “serum” OR “plasma” OR “erythrocyte” OR “red blood cell” OR “adipose tissue” OR “circulat*”) OR ("Blood"[Mesh]) OR "Serum"[Mesh]) OR "Plasma"[Mesh]) OR "Erythrocytes"[Mesh]) OR "Adipose Tissue"[Mesh]) AND (“cardiovascular disease” OR “myocardial infarction” OR “cerebrovascular disease” OR “heart disease” OR “vascular disease” OR “arterial disease” OR “carotid artery disease” OR “coronary” OR “stroke” OR “death” OR “mortalit*” OR “fatal*”) OR (“Myocardial Infarction"[Mesh] OR "Coronary Artery Disease"[Mesh] OR "Angina Pectoris"[Mesh] OR "Acute Coronary Syndrome"[Mesh] OR "Heart Failure"[Mesh] OR "Stroke"[Mesh] OR "Peripheral Arterial Disease"[Mesh] OR "Mortality"[Mesh] OR "Death"[Mesh] OR "Fatal Outcome"[Mesh]) AND (“cohort” OR “prospective” OR “nested” OR “follow up”“ OR “follow-up”“ OR “longitudinal”) OR (“Follow-Up Studies” [Mesh] OR “Prospective Studies” [Mesh] OR “Longitudinal Studies” [Mesh]).**

*Standardization of risk estimates and their uncertainty*

Log risk estimates were transformed assuming a normal distribution as described previously.[1-3] For risk estimates reported per SD, the log risk estimates and their standard errors were multiplied by 2.18. For risk estimates of comparisons of extreme quartiles or quintiles, the corresponding conversion factors were 2.18/2.54 or 2.18/2.80, respectively. One study assessing the association of erythrocyte 15:0 with incident CHD presented odds ratio and 95% CI per log (% of total fatty acids) and the lead author provided SD of the log-transformed variable to allow us to calculate odds ratio and 95% CI per SD.[4] Although the logarithmic transformation does not alter the ranking of individuals and therefore allowed the study to be included in the meta-analysis comparing high versus low exposure, we did not include it in the meta-analysis of relative risks per SD due to it being the only study evaluating 15:0 on the logarithmic scale. In addition, we conducted a sensitivity analysis evaluating top versus bottom textiles while excluding the specific study.

*Supplementary references*

1. Danesh J, Collins R, Appleby P, Peto R. Association of fibrinogen, C-reactive protein, albumin, or leukocyte count with coronary heart disease: meta-analyses of prospective studies. JAMA. 1998;279(18):1477-82. doi: 10.1001/jama.279.18.1477.

2. Chowdhury R, Warnakula S, Kunutsor S, Crowe F, Ward HA, Johnson L, et al. Association of dietary, circulating, and supplement fatty acids with coronary risk: a systematic review and meta-analysis. Ann Intern Med. 2014;160(6):398-406. doi: 10.7326/m13-1788.

3. Liang J, Zhou Q, Kwame Amakye W, Su Y, Zhang Z. Biomarkers of dairy fat intake and risk of cardiovascular disease: A systematic review and meta analysis of prospective studies. Crit Rev Food Sci Nutr. 2018;58(7):1122-30. doi: 10.1080/10408398.2016.1242114.

4. Matthan NR, Ooi EM, Van Horn L, Neuhouser ML, Woodman R, Lichtenstein AH. Plasma phospholipid fatty acid biomarkers of dietary fat quality and endogenous metabolism predict coronary heart disease risk: a nested case-control study within the Women's Health Initiative observational study. J Am Heart Assoc. 2014;3(4):e000764. doi: 10.1161/JAHA.113.000764.
