# Supplementary material for: Biomarkers of dairy fat intake, incident cardiovascular disease, and all-cause mortality: A cohort study, systematic review, and meta-analysis
Source: PLoS Med. 2021 Sep 21;18(9):e1003763. doi: 10.1371/journal.pmed.1003763 (PMC8454979; doi:10.1371/journal.pmed.1003763)

**S1 File. Supporting information tables and figures**

**Table A.** **60 YO study population characteristics at baseline by quartile of serum cholesterol ester pentadecanoic acid (15:0).^1^**

|  | Quartile of serum cholesterol ester pentadecanoic acid (15:0) | | | |  |  |
| --- | --- | --- | --- | --- | --- | --- |
|  | Q1 | Q2 | Q3 | Q4 | P^2^ | |
| Women, n (%) | 580 (56) | 571 (55) | 527 (51) | 455 (44) | <0.001 | |
|  |  |  |  |  |  | |
| Age, y | 60.5 (60.4, 60.6) | 60.5 (60.4, 60.6) | 60.4 (60.4, 60.6) | 60.4 (60.4, 60.5) | <0.001 | |
|  |  |  |  |  |  | |
| BMI, kg/m2 | 27.0 (24.4, 29.8) | 26.7 (24.1, 29.5) | 26.0 (23.7, 28.5) | 25.6 (23.7, 28.4) | <0.001 | |
|  |  |  |  |  |  | |
| Alcohol intake, g/d | 11.6 (3.7, 24.1) | 8.0 (2.7, 17.6) | 8.0 (2.7, 16.1) | 7.9 (2.2, 14.7) | <0.001 | |
|  |  |  |  |  |  | |
| Serum cholesterol ester FA, % of total FA |  |  |  |  |  | |
| Pentadecanoic acid | 0.17 (0.16, 0.18) | 0.20 (0.20, 0.21) | 0.23 (0.22, 0.24) | 0.27 (0.26, 0.29) | <0.001 | |
| Long-chain n-3 PUFA | 2.63 (2.10, 3.30) | 2.71 (2.22, 3.43) | 2.80 (2.27, 3.48) | 2.90 (2.27, 3.64) | <0.001 | |
|  |  |  |  |  |  | |
| Serum lipids (mmol/L) |  |  |  |  |  | |
| Total cholesterol | 6.2 (5.5, 6.8) | 6.0 (5.4, 6.7) | 5.8 (5.2, 6.4) | 5.7 (5.0, 6.3) | <0.001 | |
| HDL cholesterol | 1.5 (1.2, 1.8) | 1.5 (1.2, 1.7) | 1.4 (1.2, 1.7) | 1.4 (1.2, 1.7) | 0.001 | |
| LDL cholesterol | 4.0 (3.3, 4.6) | 3.9 (3.3, 4.5) | 3.8 (3.2, 4.4) | 3.7 (3.1, 4.3) | <0.001 | |
| Triglycerides | 1.3 (0.9, 1.8) | 1.2 (0.9, 1.6) | 1.1 (0.8, 1.5) | 1.0 (0.8, 1.5) | <0.001 | |
|  |  |  |  |  |  | |
| Physical activity, n (%) |  |  |  |  | 0.006 | |
| Sedentary | 136 (13) | 108 (11) | 114 (11) | 95 (9) |  | |
| Light exercise | 578 (59) | 595 (60) | 582 (59) | 555 (55) |  | |
| Moderate exercise | 199 (20) | 224 (23) | 228 (23) | 265 (26) |  | |
| Regular exercise | 70 (7) | 66 (7) | 70 (7) | 89 (9) |  | |
|  |  |  |  |  |  | |
| Smoking, n (%) |  |  |  |  | <0.001 | |
| Never | 310 (31) | 370 (37) | 424 (42) | 476 (48) |  | |
| Former | 401 (40) | 400 (41) | 379 (38) | 363 (36) |  | |
| Current | 280 (28) | 217 (22) | 197 (20) | 157 (16) |  | |
|  |  |  |  |  |  | |
| Disease prevalence, n (%) |  |  |  |  |  | |
| Type 2 diabetes | 110 (11) | 82 (8) | 60 (6) | 60 (6) | <0.001 | |
| Cardiovascular disease | 106 (10) | 97 (9) | 82 (8) | 80 (8) | 0.010 | |
| Drug-treated hypertension | 216 (21) | 216 (21) | 181 (18) | 181 (18) | 0.055 | |
| Drug-treated hyperlipidaemia | 64 (6) | 61 (6) | 43 (4) | 46 (5) | 0.085 | |
|  |  |  |  |  |  | |
| Education, n (%) |  |  |  |  | <0.001 | |
| Primary school (≤9 y) | 347 (35) | 308 (31) | 244 (24) | 247 (25) |  | |
| Secondary school (≤12 y) | 443 (44) | 444 (45) | 453 (45) | 405 (41) |  | |
| University or college (>12 y) | 210 (21) | 243 (24) | 301 (30) | 347 (35) |  | |
|  |  |  |  |  |  | |
| Vegetable intake^3^, n (%) |  |  |  |  | 0.011 | |
| ≥1 time per day | 604 (60) | 648 (65) | 672 (67) | 660 (65) |  | |
| <1 time per day | 402 (40) | 356 (35) | 335 (33) | 348 (35) |  | |
|  |  |  |  |  |  | |
| Fruit and berry intake, n (%) |  |  |  |  | 0.002 | |
| ≥1 time per day | 598 (60) | 638 (64) | 662 (66) | 678 (67) |  | |
| <1 time per day | 407 (40) | 366 (36) | 345 (34) | 332 (33) |  | |
|  |  |  |  |  |  | |
| Lean fish intake, n (%) |  |  |  |  | 0.028 | |
| ≥1 time per week | 636 (63) | 676 (67) | 675 (67) | 701 (69) |  | |
| <1 time per week | 369 (37) | 327 (33) | 329 (33) | 308 (31) |  | |
|  |  |  |  |  |  | |
| Oily fish intake, n (%) |  |  |  |  | 0.350 | |
| ≥1 time per week | 241 (24) | 249 (25) | 245 (24) | 274 (27) |  | |
| <1 time per week | 760 (76) | 755 (75) | 761 (76) | 733 (73) |  | |
|  |  |  |  |  |  | |
| Processed meat intake (as main dish), n (%) |  |  |  |  | <0.001 | |
| ≥1 time per week | 443 (44) | 395 (39) | 364 (36) | 346 (34) |  | |
| <1 time per week | 561 (56) | 609 (61) | 641 (64) | 662 (66) |  | |
|  |  |  |  |  |  | |
| Bread intake^4^, n (%) |  |  |  |  | <0.001 | |
| >2 servings per day | 427 (42) | 460 (46) | 513 (51) | 565 (56) |  | |
| ≤2 servings per day | 579 (58) | 542 (54) | 491 (49) | 441 (44) |  | |

^1^ Values are median (25^th^, 75^th^ percentiles) or n (%). FA, fatty acid; HDL, high-density lipoprotein; LDL, low-density lipoprotein; PUFA, polyunsaturated fatty acids. ^2^For continuous variables (i.e., age, BMI, alcohol intake, and serum fatty acids and lipids), trend across serum 15:0 quartiles were assessed in linear regression models with quartile medians as the only independent variable. For categorical variables (i.e., physical activity, education, disease prevalence, and dietary intakes), differences in frequencies between quartiles were assessed with χ^2^ tests. ^3^Includes root vegetables.^4^One sandwich per serving.

**Table B.** **Hazard ratios of primary (incident CVD and all-cause mortality) and secondary outcomes (incident CHD, stroke and CVD mortality) with serum 15:0 evaluated per interquintile range, per SD, or per % of totals fatty acids in 60 YO study.^1^**

| Outcome | Unit | HR (95% CI)^2^ |
| --- | --- | --- |
| Incident CVD^3^ | IQR^4^ | 0.75 (0.61, 0.93) |
|  | SD^5^ | 0.89 (0.81, 0.97) |
|  | % of total fatty acids | 0.08 (0.01, 0.53) |
|  |  |  |
| All-cause mortality | IQR | 0.91 (0.74, 1.12) |
|  | SD | 0.96 (0.88, 1.05) |
|  | % of total fatty acids | 0.44 (0.07, 2.79) |
|  |  |  |
| Incident CHD | IQR | 0.70 (0.54, 0.91) |
|  | SD | 0.86 (0.77, 0.96) |
|  | % of total fatty acids | 0.04 (0.00, 0.44) |
|  |  |  |
| Incident stroke | IQR | 0.87 (0.61, 1.25) |
|  | SD | 0.94 (0.81, 1.10) |
|  | % of total fatty acids | 0.28 (0.01, 7.38) |
|  |  |  |
| CVD mortality | IQR | 0.89 (0.63, 1.25) |
|  | SD | 0.95 (0.82, 1.10) |
|  | % of total fatty acids | 0.35 (0.02, 7.61) |

^1^CVD, cardiovascular disease; HR, hazard ratio; IQR, interquintile range; SD, standard deviation.^2^ Hazard ratios were estimated using Cox proportional hazard models adjusted for age, sex, BMI, alcohol intake, smoking habits, physical activity, education, and prevalent hypertension, hyperlipidaemia and type 2 diabetes. Models used for evaluating all-cause or CVD mortality were further adjusted for prevalent CVD. ^3^Participants with prevalent CVD at baseline were excluded from analyses on incident CHD and stroke. ^4^IQR=0.11% of total fatty acids. ^5^SD=0.05% of total fatty acids.

**Table C.** **Hazard ratios of incident CHD, stroke and CVD mortality per interquintile range of serum pentadecanoic acid (15:0) in 60 YO study.^1^**

| Outcome |  | Model^2^ |  | P_linear_^3^ | P_nonlinear_^4^ |
| --- | --- | --- | --- | --- | --- |
| Incident CHD^5^ | Cases/Person-years |  | 386/55 832 |  |  |
|  | HR (95% CI)^6^ | 1 | 0.71 (0.55, 0.92) | 0.009 | 0.95 |
|  |  | 2 | 0.61 (0.48, 0.79) | <0.001 | 0.93 |
|  |  | 3 | 0.70 (0.54, 0.91) | 0.008 | 0.66 |
| Incident stroke^5^ | Cases/Person-years |  | 192/55 832 |  |  |
|  | HR (95% CI) | 1 | 0.75 (0.52, 1.09) | 0.13 | 0.17 |
|  |  | 2 | 0.71 (0.49, 1.03) | 0.07 | 0.17 |
|  |  | 3 | 0.87 (0.61, 1.25) | 0.45 | 0.47 |
| CVD mortality^5^ | Cases/Person-years |  | 198/64 605 |  |  |
|  | HR (95% CI) | 1 | 0.71 (0.50, 1.02) | 0.06 | 0.59 |
|  |  | 2 | 0.67 (0.47, 0.95) | 0.025 | 0.75 |
|  |  | 3 | 0.89 (0.63, 1.25) | 0.51 | 0.72 |

^1^CI, confidence interval; CVD, cardiovascular disease; HR, hazard ratio.^2^Model 1: includes serum 15:0 as only covariate and was thus used to assess crude associations. Model 2 included adjustments for age and sex. Model 3 was further adjusted for BMI, alcohol intake, smoking habits, physical activity, education, and prevalent hypertension, hyperlipidaemia and type 2 diabetes. ^3^Linear associations were evaluated per interquintile ranges (i.e., midpoints of the first and fifth quintiles) of biomarker 15:0. ^4^Non-linear trends were evaluated using restricted cubic splines (knots at 10^th^, 50^th^, and 90^th^ percentiles). ^5^Participants with prevalent CVD at baseline were excluded from analyses on incident CHD, stroke and CVD mortality. ^6^Hazard ratios were estimated using Cox proportional hazard models.

**Table D**. **Hazard ratios (95% CI) of incident CVD and all-cause mortality by serum pentadecanoic acid (15:0) (per 1 IQR-increase) according to sex, BMI, and serum proportions of long-chain n-3 PUFA in 60 YO study.^1^**

|  |  | Sex | |  | BMI | |  | Serum EPA+DHA | |
| --- | --- | --- | --- | --- | --- | --- | --- | --- | --- |
| Outcome |  | **Women** | **Men** |  | **< Median** | **≥ Median** |  | **< Median** | **≥ Median** |
| Incident CVD^2^ | N | 1,994 | 1,791 |  | 1,887 | 1,898 |  | 1,892 | 1,893 |
|  | HR (95% CI)^3^ | 0.68 (0.46, 1.01) | 0.79 (0.61, 1.02) |  | 0.96 (0.71, 1.31) | 0.83 (0.63, 1.08) |  | 0.97 (0.75, 1.27) | 0.82 (0.60, 1.13) |
|  | P_interaction_^4^ | 0.51 | |  | 0.58 | |  | 0.88 | |
| All-cause mortality | N | 2,133 | 2,017 |  | 2,077 | 2,073 |  | 2,075 | 2,075 |
|  | HR (95% CI) | 0.93 (0.65, 1.32) | 0.90 (0.70, 1.16) |  | 0.97 (0.73, 1.28) | 1.06 (0.80, 1.39) |  | 0.87 (0.66, 1.15) | 1.25 (0.96, 1.64) |
|  | P_interaction_ | 0.88 | |  | 0.38 | |  | 0.11 | |

^1^BMI, body mass index; CVD, cardiovascular disease; DHA, docosahexaenoic acid; EPA, eicosapentaenoic acid; IQR, interquintile range; PUFA, polyunsaturated fatty acid. ^2^Participants with prevalent CVD at baseline were excluded from analyses on incident CVD. ^3^Sub-group specific hazard ratios were estimated in multivariable-adjusted models (including sex, age, BMI, physical activity, alcohol intake, smoking habits, and comorbidity as covariates). ^4^Interactions of each potential effect-modificator (sex, BMI, and serum n-3 PUFA) and serum 15:0 were evaluated in multivariable-adjusted models with a cross-product term of serum 15:0 and the potential effect modificator.

**Table E.** **Hazard ratios (95% CI) of incident CVD and all-cause mortality by serum pentadecanoic acid (15:0) (per 1 IQR-increase) assessed in sensitivity analyses excluding early cases, censoring at 10 years of follow-up or by excluding individuals with prevalent CVD at baseline in 60 YO study.^1^**

| Outcome |  | N | Median serum 15:0  (10^th^, 90^th^ percentiles) | Cases / Person-years | HR (95% CI) |
| --- | --- | --- | --- | --- | --- |
| Incident CVD^2^ | Main analysis^3^ | 3,785 | 0.22 (0.17, 0.28) | 578 / 55,832 | 0.75 (0.61, 0.93) |
|  | Additional adjustment for self-reported dietary habits^4^ | 3,785 | 0.22 (0.17, 0.28) | 578 / 55,832 | 0.76 (0.62, 0.94) |
|  | Exclude cases ≤ 2 years after baseline | 3,725 | 0.22 (0.17, 0.28) | 518 / 55,771 | 0.80 (0.64, 0.99) |
|  | Censor after 10 years of follow-up | 3,785 | 0.22 (0.17, 0.28) | 320 / 35,647 | 0.69 (0.51, 0.93) |
| All-cause mortality | Main analysis^3^ | 4,150 | 0.22 (0.17, 0.28) | 676 / 64,605 | 0.91 (0.74, 1.12) |
|  | Additional adjustment for self-reported dietary habits^4^ | 4,150 | 0.22 (0.17, 0.28) | 676 / 64,605 | 0.94 (0.77, 1.14) |
|  | Exclude deaths ≤ 2 years after baseline | 4,121 | 0.22 (0.17, 0.28) | 647 / 64,571 | 0.92 (0.74, 1.13) |
|  | Censor after 10 years of follow-up | 4,150 | 0.22 (0.17, 0.28) | 298 / 40,291 | 0.92 (0.68, 1.25) |
|  | Exclude individuals with prevalent CVD | 3,785 | 0.22 (0.17, 0.28) | 568 / 59,309 | 0.85 (0.67, 1.07) |

^1^ CVD, cardiovascular disease; IQR, interquintile range. **^2^**Participants with prevalent CVD at baseline were excluded from analyses on incident CVD.^3^ Model 3, adjusted for age, sex, BMI, alcohol intake, smoking habits, physical activity, education, and prevalent hypertension, hyperlipidaemia, type 2 diabetes, and (for evaluation of all-cause mortality) cardiovascular disease. ^4^Model 3 with additional adjustment for self-reported intake of vegetables (“>1 serving per day”; “almost daily or daily”; “a couple of times per week or less”; or “never”), fruits and berries (“several times per day”; “daily”; “a couple of times per week”; or “seldom”), lean fish (“≥3 times per week”, “1-2 times per week”, “seldom”), oily fish (“≥3 times per week”, “1-2 times per week”, “seldom”), and processed meat as main dish (“a couple of times per week”, “once a week”, “a couple of times per month”, or “almost never”) .

**Table F. Characteristics of studies included in the systematic review.**

| **Study [Year]** | **Country** | **Study design** | **Inclusion criteria** | **Exclusion criteria** | **Primary outcomes** | **Secondary outcomes** | **Dairy fat biomarkers** | **Follow-up years** |
| --- | --- | --- | --- | --- | --- | --- | --- | --- |
| Warensjö (2004) | Sweden | NCC | Cases registered in the northern Sweden MONICA incidence registry, and participated in health surveys before the AMI event and at the same time had donated a blood sample to the Northern Sweden Medical Research Bank. | Uncertain history of AMI, stroke or cancer. Cases deceased or moved from the area before end of follow-up (30 September 1994) were also excluded. | AMI incidence | Nil | 15:0, 17:0, and their sum in serum cholesterol esters and phospholipids. | Mean: 1.5  Median: 1.25 |
| Sun (2007) | USA | NCC | Participants who provided a blood sample, and are free of diagnosed cancer and cardiovascular disease at the time of blood drawing. | Missing fatty acid concentration data. | Total CHD defined as nonfatal incident AMI or CHD mortality. | Nil | 15:0, 17:0, and *t*16:1n-7 in total plasma and erythrocytes. | Max: 7.5 |
| Yamagishi (2008) | USA | Cohort | Participants in the Minneapolis field centre arm of the ARIC cohort who had stored plasma sample saved at baseline. | Prior CHD, stroke or HF at baseline survey, those without plasma fatty acid data; or non-white subjects due to their small number. | Heart failure incidence | Nil | 15:0 in plasma cholesterol esters and phospholipids. | Median: 14.3 |
| Warensjö (2009) | Sweden | NCC | VIP or MONICA participants with baseline blood sample who experienced a stroke before the age of 75. | Prior AMI, stroke, or cancer. | Stroke incidence | Nil | 15:0, 17:0, and their sum in serum cholesterol esters and phospholipids. | Mean: 3 |
| Warensjö (2010) | Sweden | NCC | VIP participants with AMI and baseline blood samples for fatty acid analyses. | Prior AMI, stroke, or cancer. | AMI incidence | Nil | 15:0, 17:0, and their sum in serum phospholipids. | Mean: 3.1 (women), 3.9 (men) |
| Khaw (2012) | UK | NCC | EPIC-Norfolk participants with available measurement of plasma phospholipid fatty acids. | Not reported. | CHD incidence | Nil | 15:0, 17:0, and their sum in plasma phospholipids. | Mean: 13 |
| Malik (2012) | USA | NCC | HPFS participants with baseline blood samples. | Not reported. | CHD incidence | Nil | 15:0, 17:0, and *t*16:1n-7 in total plasma and erythrocytes. | . |
| Otto (2013) | USA | Cohort | MESA participants with baseline plasma phospholipid fatty acids. | Missing incident CVD data. | CVD and CHD incidence | CVD events excluding cases of angina pectoris. | 15:0 and *t*16:1n-7 in plasma phospholipids. | Mean: 7.0 |
| Matsumoto (2013) | USA | NCC | PHS participants with HF and baseline blood samples. | Not reported. | Heart failure incidence | Nil | 15:0 in plasma phospholipids. | Mean: 10 |
| Tokede (2013) | USA | NCC | PHS participants with HF and baseline blood samples. | Not reported. | Heart failure incidence | Nil | *t*16:1n-7 in plasma phospholipids | Mean: 10 |
| Yaemsiri (2013) | USA | NCC | WHI participants with confirmed ischaemic stroke. | Insufficient baseline blood quantity to analyse fatty acid content. | Stroke incidence | Nil | 15:0 and 17:0 in plasma phospholipids. | Max: 10 |
| Yamagishi (2013) | USA | Cohort | Participants in the Minneapolis field centre arm of the ARIC cohort who had stored plasma sample saved at baseline. | Prior CHD, stroke or HF at baseline survey, those without plasma fatty acid data; or non-white subjects due to their small number. | Stroke incidence | Nil | 15:0 plasma cholesterol esters and phospholipids. | Median: 19.9  Max: 22.4 |
| Matthan (2014) | USA | NCC | WHI participants with confirmed cases of CHD (hospitalized myocardial infarction [MI], definite silent MI, and coronary death defined as death consistent with CHD as the underlying cause, based on review of medical records and death certificate) from the September 2005 database. | Cases were excluded based on: (1) lack of available baseline plasma sample, (2) lack of baseline food frequency questionnaire, and (3) CVD reported at baseline. Potential controls were excluded for all these reasons as well as CVD occurring during follow-up (mean 4.5 years). | CHD incidence and mortality | Nil | 15:0 in plasma phospholipids. | Mean: 4.5 |
| Yakoob (2014) | USA | NCC | Subjects from the HPFS and NHS who had blood samples taken at baseline and were free of prevalent CVD or cancer. | Missing fatty acid concentration data. | Stroke incidence | Stroke subtype - ischaemic or haemorrhagic | 15:0, 17:0, and *t*16:1n-7 in total plasma and erythrocytes. | Median: 8.3 |
| Iggman (2016) | Sweden | Cohort | ULSAM cohort with adipose tissue biopsy specimens obtained between 1991 and 1995. | Not reported. | Cardiovascular and all-cause mortality | Nil | 15:0 and 17:0 in adipose tissue. | Median: 14.8 |
| Otto (2018) | USA | Cohort | CHS participants who had plasma phospholipid fatty acids measures available at baseline and free of CVD. | Not reported. | Total mortality, cause-specific mortality (CVD, CHD, stroke, and non-CVD mortality), incident CVD, and CVD subtypes. | Subcategories of non-CVD mortality. | 15:0, 17:0, and *t*16:1n-7 in plasma phospholipids. | Mean: 12.9 |
| Laursen (2018) | Denmark | Case-Cohort | Participants in the DCH cohort who were identified as having a stroke. | Participants with prior stroke or cancer, and those for whom exposure or covariate information was missing. | Stroke incidence | Stroke subtype - ischaemic, intracerebral haemorrhage, or subarachnoid haemorrhage | 15:0 and 17:0 in adipose tissue. | Median: 12.8 |
| 60YO | Sweden | Cohort | Participants living in Stockholm county, who turned 60 between July 1 1997 and June 30 1998. | Those without fasting blood samples at baseline or follow-up information until December 31, 2014. | CVD incidence, all-cause mortality | CVD mortality, coronary, incident heart disease, incident ischaemic stroke | 15:0 in serum cholesterol ester. | Median: 16.6 |

AMI acute myocardial infarction; ARIC Atherosclerosis Risk in Communities; CHS Cardiovascular Health Study; CVD cardiovascular disease; DCH Danish Diet, Cancer, and Health cohort; EPIC European Prospective Investigation into Cancer; HF heart failure; HPFS Health Professionals Follow-up Study; MESA Multi-Ethnic Study of Atherosclerosis; MONICA the northern Sweden Monitoring of Trends and Determinants in Cardiovascular disease; NCC nested case-control study; NHS Nurses’ Health Study; PHS Physician's Health Study; ULSAM Uppsala Longitudinal Cohort of Adult Men; VIP Västerbotten Intervention Program; WHI Women’s Health Initiative.

**Table G. Newcastle-Ottawa Score (NOS) calculation for studies included in the systematic review.**

| **STUDY** | **SELECTION** | | | | **COMPARABILITY** | **OUTCOME** | | | **NOS SCORE** |
| --- | --- | --- | --- | --- | --- | --- | --- | --- | --- |
|  | 1. Representativeness of the exposed cohort.^1^ | 2. Selection of the non-exposed cohort.^2^ | 3. Ascertainment of exposure.^3^ | 4. Demonstration that outcome of interest was not present at the start of the study.^4^ | 1. Comparability of cohorts on the basis of the design or analysis.^5^ | 1. Assessment of outcome.^6^ | 2. Was follow-up long enough for outcomes to occur?^7^ | 3. Adequacy of follow-up of cohorts.^8^ |  |
| **Warensjö (2004)** | 1 | 1 | 1 | 1 | 1 | 1 | 1 | 1 | 8 |
| **Sun (2007)** | 0 | 1 | 1 | 1 | 1 | 1 | 1 | 1 | 7 |
| **Yamagishi (2008)** | 1 | 1 | 1 | 1 | 2 | 1 | 1 | 1 | 9 |
| **Warensjö (2009)** | 1 | 1 | 1 | 0 | 1 | 1 | 1 | 1 | 7 |
| **Warensjö (2010)** | 1 | 1 | 1 | 1 | 2 | 1 | 1 | 1 | 9 |
| **Khaw (2012)** | 1 | 1 | 1 | 1 | 2 | 1 | 1 | 1 | 9 |
| **Malik (2012)** | 1 | 1 | 1 | 1 | 0 | 1 | 1 | 1 | 7 |
| **Otto (2013)** | 1 | 0 | 1 | 1 | 2 | 1 | 1 | 1 | 8 |
| **Matsumoto (2013)** | 0 | 1 | 0 | 0 | 1 | 1 | 1 | 1 | 5 |
| **Tokede (2013)** | 0 | 1 | 1 | 0 | 1 | 1 | 1 | 1 | 6 |
| **Yaemsiri (2013)** | 1 | 1 | 1 | 1 | 1 | 1 | 1 | 1 | 8 |
| **Yamagishi (2013)** | 1 | 1 | 1 | 1 | 2 | 1 | 1 | 1 | 9 |
| **Matthan (2014)** | 1 | 1 | 1 | 0 | 2 | 1 | 1 | 1 | 8 |
| **Yakoob (2014)** | 0 | 1 | 1 | 0 | 2 | 1 | 1 | 1 | 7 |
| **Iggman (2016)** | 1 | 1 | 1 | 1 | 1 | 1 | 1 | 1 | 8 |
| **Otto (2018)** | 1 | 1 | 1 | 1 | 2 | 0 | 1 | 1 | 8 |
| **Laursen (2018)** | 1 | 1 | 1 | 1 | 2 | 1 | 1 | 1 | 9 |
| **60YO (2020)** | 1 | 1 | 1 | 1 | 2 | 1 | 1 | 1 | 9 |

^1^Truly representative of the population most at risk of CVD in the community [1]; somewhat representative of the population most at risk of CVD in the community [1]; selected group of users e.g. nurses, volunteers, physicians. [0]; no description of the derivation of the cohort [0]. ^2^Drawn from the same community or population as the exposed cohort [1]; drawn from a different source [0]; no description [0] .^3^Secure record e.g. surgical or medical records, death certificates... [1]; structured interview [1]; written self-report [0]; no description [0]. ^4^Yes [1]; no [0]. ^5^Study controls for the most important confounding factors [1]; study controls for any additional confounding factors [1]. ^6^Independent blind assessment [1]; record linkage [1]; self-report [0]; no description [0]. ^7^Yes (≥ 12 months) [1]; no [0]. ^8^Complete follow-up; all subjects accounted for [1]; subjects lost to follow-up unlikely to introduce bias; small numbers lost; > 75% follow-up, or description provided of those lost [1]; follow-up rate <75%) and no description of those lost [0]; no statement [0]

**Table H.** **Pooled risk estimates of cardiovascular disease (CVD) subtypes and all-cause mortality per standard deviation (SD) increase in 15:0, 17:0, and *t*16:1n-7.^1^**

|  | **Studies (n)** | **Cases (n)** | **Risk estimate (95% CI)** | **I^2^ (%)** |
| --- | --- | --- | --- | --- |
| **15:0** | | | | |
| Total CVD | 12 | 7,680 | 0.93 (0.86, 1.00) | 58.6 |
| CVD incidence | 3 | 2,068 | 0.86 (0.66, 1.11) | 88.3 |
| CVD mortality | 3 | 1,282 | 1.03 (0.97, 1.09) | 0.0 |
| CHD incidence | 8 | 4,909 | 0.91 (0.83, 1.00) | 67.3 |
| CHD mortality | 1 | 567 | 1.06 (0.98, 1.15) | - |
| Stroke incidence | 4 | 1,793 | 1.00 (0.93, 1.09) | 18.1 |
| Stroke mortality | 1 | 188 | 1.04 (0.90, 1.21) | - |
| HF incidence | 1 | 788 | 0.91 (0.77, 1.08) | - |
| All-cause mortality | 3 | 2,068 | 0.86 (0.66, 1.11) | 88.3 |
| **17:0** | | | | |
| Total CVD | 9 | 6,131 | 0.93 (0.88, 0.98) | 17.5 |
| CVD incidence | 1 | 1,301 | 0.95 (0.89, 1.01) | - |
| CVD mortality | 2 | 1,084 | 0.92 (0.85, 1.00) | 0.0 |
| CHD incidence | 6 | 4,383 | 0.93 (0.87, 0.98) | 12.0 |
| CHD mortality | 1 | 567 | 0.93 (0.84, 1.02) | - |
| Stroke incidence | 4 | 2,195 | 0.93 (0.81, 1.07) | 48.1 |
| Stroke mortality | 1 | 188 | 0.81 (0.68, 0.97) | - |
| HF incidence | 0 | 0 | - | - |
| All-cause mortality | 2 | 3,033 | 0.96 (0.85, 1.08) | 73.4 |
| ***t*16:1n-7** | | | | |
| Total CVD | 6 | 4,126 | 1.00 (0.96, 1.05) | 0.0 |
| CVD incidence | 2 | 1,490 | 1.01 (0.96, 1.07) | 0.0 |
| CVD mortality | 1 | 833 | 1.01 (0.94, 1.08) | - |
| CHD incidence | 5 | 2,870 | 1.03 (0.97, 1.09) | 0.0 |
| CHD mortality | 1 | 567 | 1.04 (0.95, 1.14) | - |
| Stroke incidence | 1 | 529 | 1.05 (0.96, 1.15) | - |
| Stroke mortality | 1 | 188 | 0.93 (0.79, 1.09) | - |
| HF incidence | 1 | 788 | 0.91 (0.80, 1.04) | - |
| All-cause mortality | 1 | 2,428 | 1.03 (0.99, 1.08) | - |

^1^ CHD, coronary heart disease; CVD, cardiovascular disease; HF, heart failure.

**Table I: Risk estimates of total cardiovascular disease (CVD) comparing top versus bottom tertile of 15:0, 17:0 and *t*16:1n-7 in subgroups by age, sex, duration of follow-up, or study location.**

|  | **Studies (n)** | **Cases (n)** | **Risk estimate (95% CI)** | **I^2^ (%)** |
| --- | --- | --- | --- | --- |
| **15:0** | | | | |
| Baseline age (y) | | | | |
| < 61 | 9 | 4,617 | 0.77 (0.69, 0.85) | 0.0 |
| $\text{≥}$ 61 | 8 | 7,333 | 0.98 (0.86, 1.12) | 38.1 |
| *P_heterogeneity_^1^* |  |  | 0.01 |  |
| Sex | | | | |
| Male | 7 | 2,356 | 0.88 (0.75, 1.02) | 0.0 |
| Female | 7 | 3,153 | 0.87 (0.71, 1.07) | 20.9 |
| *P_heterogeneity_^1^* |  |  | 0.95 |  |
| Follow up duration (y) | | | | |
| < 10 | 8 | 3,732 | 0.83 (0.67, 1.03) | 33.4 |
| $\text{≥}$ 10 | 8 | 7,760 | 0.90 (0.77, 1.06) | 74.3 |
| *P_heterogeneity_^1^* |  |  | 0.56 |  |
| Study location | | | | |
| USA | 10 | 6,028 | 0.95 (0.84, 1.08) | 31.4 |
| Europe | 7 | 5,922 | 0.81 (0.69, 0.94) | 40.2 |
| *P_heterogeneity_^1^* |  |  | 0.13 |  |
| **17:0** | | | | |
| Age (y) | | | | |
| < 61 | 6 | 3,089 | 0.79 (0.68, 0.90) | 2.4 |
| $\text{≥}$ 61 | 6 | 5,920 | 0.90 (0.81, 1.00) | 0.0 |
| *P_heterogeneity_^1^* |  |  | 0.16 |  |
| Sex | | | | |
| Male | 5 | 1,203 | 1.00 (0.78, 1.27) | 0.0 |
| Female | 5 | 1,716 | 0.67 (0.41, 1.11) | 51.8 |
| *P_heterogeneity_^1^* |  |  | 0.40 |  |
| Follow up duration (y) | | | | |
| < 10 | 6 | 2,325 | 0.77 (0.61, 0.96) | 12.2 |
| $\text{≥}$ 10 | 5 | 6,226 | 0.86 (0.79, 0.94) | 0.0 |
| *P_heterogeneity_^1^* |  |  | 0.37 |  |
| Study location | | | | |
| USA | 6 | 3,659 | 0.90 (0.80, 1.01) | 0.0 |
| Europe | 6 | 5,350 | 0.81 (0.71, 0.93) | 8.9 |
| *P_heterogeneity_^1^* |  |  | 0.30 |  |
| ***t*16:1n-7** | | | | |
| Baseline age (y) | | | | |
| < 61 | 2 | 954 | 0.84 (0.65, 1.09) | 0.0 |
| $\text{≥}$ 61 | 4 | 2,523 | 1.05 (0.94, 1.18) | 0.0 |
| *P_heterogeneity_^1^* |  |  | 0.20 |  |
| Sex | | | | |
| Male | 3 | 1368 | 1.07 (0.70, 1.65) | 56.5 |
| Female | 2 | 619 | 0.97 (0.65, 1.45) | 0.0 |
| *P_heterogeneity_^1^* |  |  | 0.82 |  |
| Follow up duration (y) | | | | |
| < 10 | 3 | 930 | 1.05 (0.81, 1.36) | 0.0 |
| $\text{≥}$ 10 | 2 | 2,089 | 0.98 (0.87, 1.11) | 50.3 |
| *P_heterogeneity_^1^* |  |  | 0.64 |  |
| Study location | | | | |
| USA | 6 | 4,701 | 1.01 (0.91, 1.12) | 0.0 |
| Europe | 0 | - | - | - |
| *P_heterogeneity_^1^* |  |  | - |  |

^1^Heterogeneity by baseline age ( < 60 y vs. $\text{≥}$ 60 y), sex (male vs female), follow up duration (< 10 y vs $\text{≥}$ 10 y), or study location (USA vs Europe) was assessed by meta-regression random-effects meta-regression.

**Fig A.** Relationship between the dairy intake score and pentadecanoic acid in serum cholesterol esters, evaluated using restricted cubic splines and adjusted for age, sex, BMI, physical activity, alcohol use, and smoking status in 60 YO study. The circles represent the point estimates and the error bars, 95% CIs. The dairy intake score was based on self-reported habits regarding use of butter, cheese, milk, and yoghurt.[1] The histogram shows the distribution of the dairy intake score in the cohort.


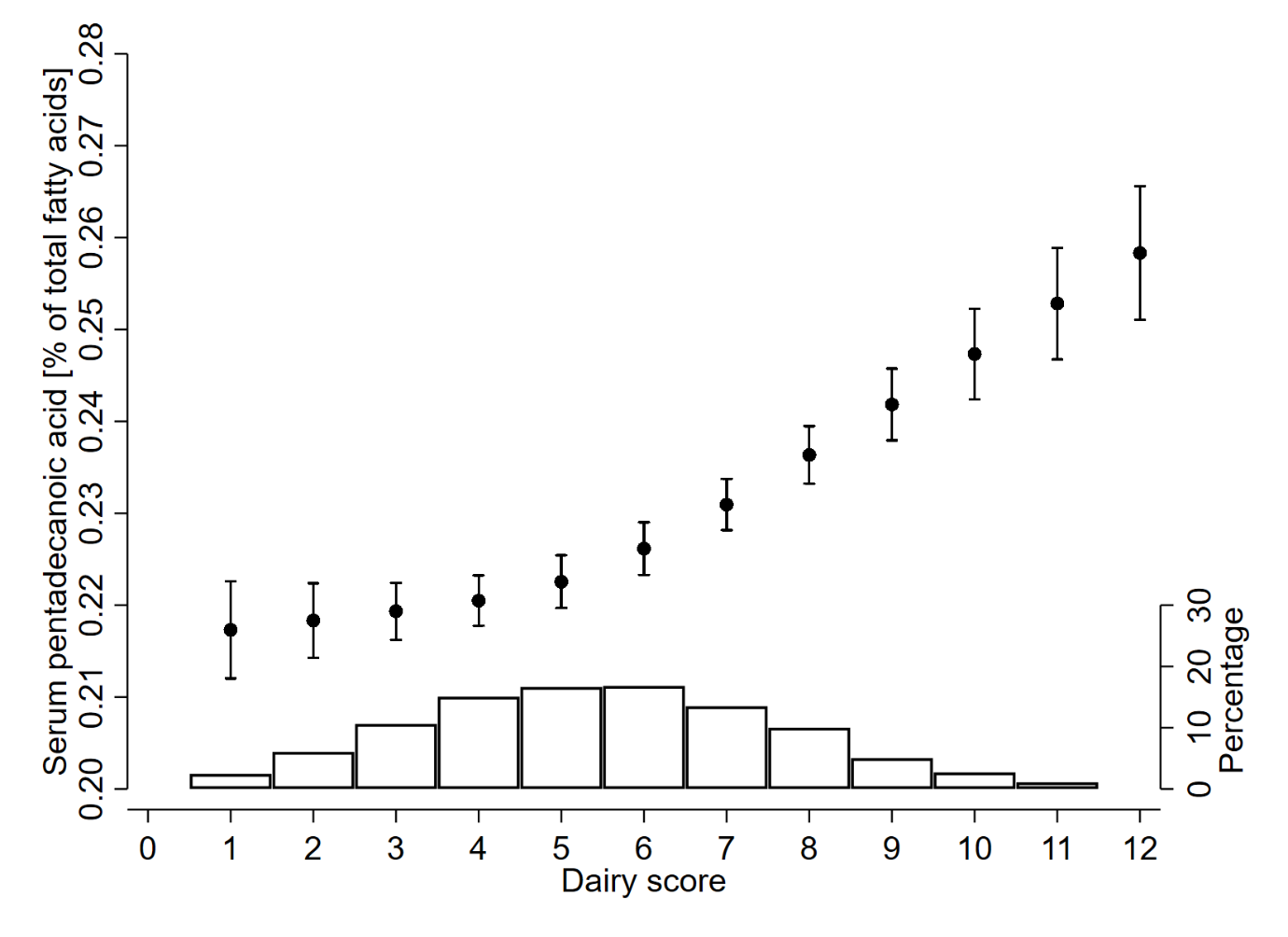
*Supplementary references*

1. Laguzzi F, Alsharari Z, Risérus U, Vikström M, Sjögren P, Gigante B, et al. Cross-sectional relationships between dietary fat intake and serum cholesterol fatty acids in a Swedish cohort of 60-year-old men and women. J Hum Nutr Diet,. 2016;29(3):325-37. doi: 10.1111/jhn.12336.

**Fig B.** **Funnel plot of studies included in the meta-analysis for serum 15:0 (A), 17:0 (B), and *t*16n-7 (C)**

A


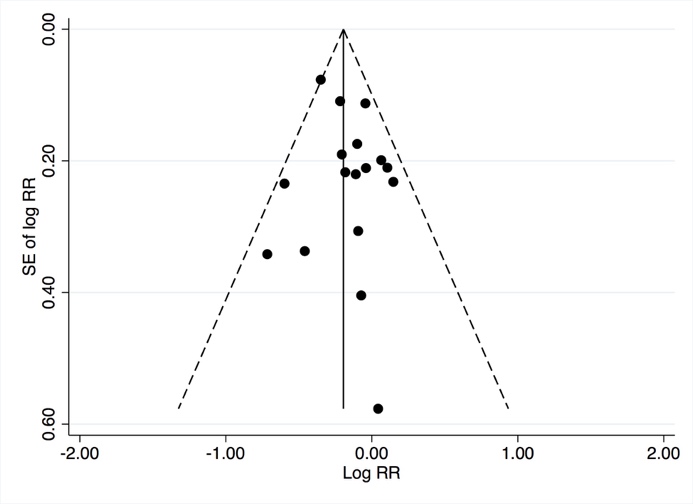


B


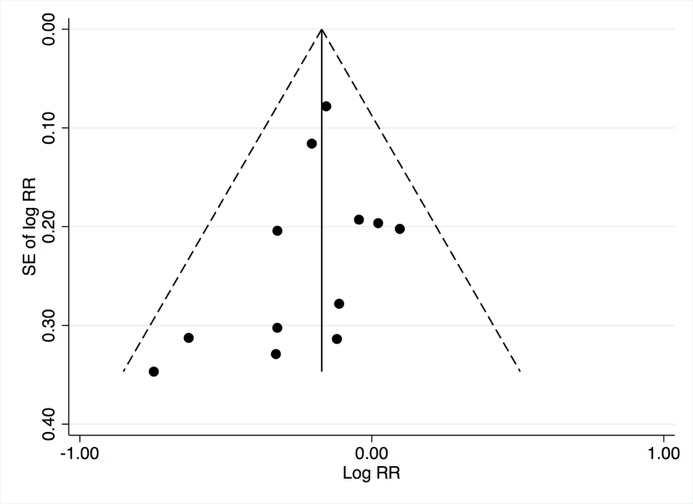


C


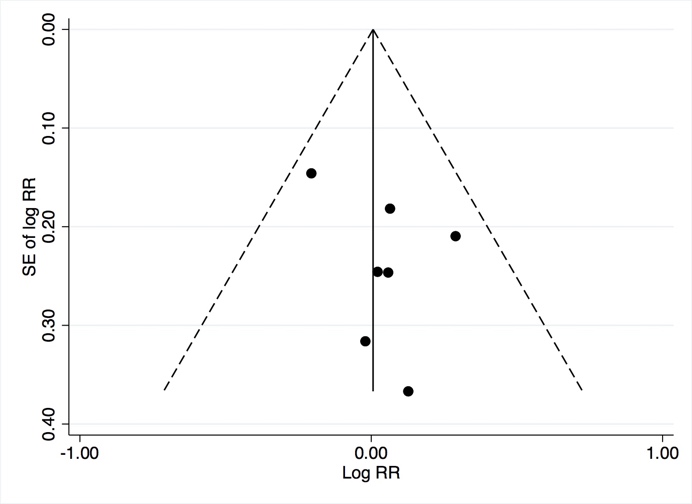

Supplement: S1 File — Table A. 60YO study population characteristics at baseline by quartile of serum cholesterol ester pentadecanoic acid (15:0). Table B. Hazard ratios of primary (incident CVD and all-cause mortality) and secondary outcomes (incident CHD, stroke, and CVD mortality) with serum 15:0 evaluated per interquintile range, per SD, or per % of totals fatty acids in the 60YO study. Table C. Hazard ratios of incident CHD, stroke, and CVD mortality per interquintile range of serum pentadecanoic acid (15:0) in the 60YO study. Table D. Hazard ratios (95% CI) of incident CVD and all-cause mortality by serum pentadecanoic acid (15:0) (per 1 IQR increase) according to sex, BMI, and serum proportions of long-chain n-3 PUFA in the 60YO study. Table E. Hazard ratios (95% CI) of incident CVD and all-cause mortality by serum pentadecanoic acid (15:0) (per 1 IQR increase) assessed in sensitivity analyses excluding early cases, censoring at 10 years of follow-up or by excluding individuals with prevalent CVD at baseline in the 60YO study. Table F. Characteristics of studies included in the systematic review. Table G. Newcastle–Ottawa Score (NOS) calculation for studies included in the systematic review. Table H. Pooled risk estimates of cardiovascular disease (CVD) subtypes and all-cause mortality per standard deviation (SD) increase in 15:0, 17:0, and t16:1n-7. Table I. Risk estimates of total cardiovascular disease (CVD) comparing top versus bottom tertile of 15:0, 17:0, and t16:1n-7 in subgroups by age, sex, duration of follow-up, or study location. Fig A. Relationship between the dairy intake score and pentadecanoic acid in serum cholesterol esters, evaluated using restricted cubic splines and adjusted for age, sex, BMI, physical activity, alcohol use, and smoking status in the 60YO study. The circles represent the point estimates and the error bars, 95% CIs. The dairy intake score was based on self-reported habits regarding use of butter, cheese, milk, and yoghurt [1]. The histogram show [file pmed.1003763.s006.docx]
