# Supplementary material for: Biomarkers of dairy fat intake, incident cardiovascular disease, and all-cause mortality: A cohort study, systematic review, and meta-analysis
Source: PLoS Med. 2021 Sep 21;18(9):e1003763. doi: 10.1371/journal.pmed.1003763 (PMC8454979; doi:10.1371/journal.pmed.1003763)
Supplement: Attachment — Submitted filename: Response to reviewers and editors.docx [file pmed.1003763.s007.docx]

***Response to reviewers and editors***

| **Reviewer comment** | **Authors’ response** | **Modifications to the manuscript** |
| --- | --- | --- |
| **Editor** | | |
| Noting PLOS' data policy, https://journals.plos.org/plosmedicine/s/data-availability, please adapt your data statement (submission form) so that the point of contact for inquiries about data is not an author, and preferably not an individual | Thank you, we have now modified the data availability statement and will submit the revised statement during resubmission | Page 17, the data sharing information has been removed. The following sentence about data availability will be included in the article meta data: “Data from the systematic review are within the manuscript and supporting Information files. Due to legal restrictions, some access restrictions apply to the remaining data underlying the findings. Requests could be sent to Karolinska Institutet, Contact details: Karolinska Institutet, 171 77 Stockholm, Sweden, Telephone: +46 8 524 800” |
| At the start of the abstract, we suggest adding an introductory sentence, say, to state the study's aim. | We have modified this accordingly | Page 3, edited: “We *aimed to* investigate the association… “ |
| Please quote aggregate demographic details for participants in the cohort study in your abstract | We have aggregated the demographic details | Page 3, edited: “We measured 15:0 in serum cholesterol esters at baseline in *4150 Swedish adults (median age 60.5 years, 51% female)*.” |
| Please adapt your abstract by adding a new final sentence to the "Methods and findings" subsection, which should begin "Study limitations include ..." or similar and should quote 2-3 of the study's main limitations. | We have added the main study limitations in the abstract as suggested | Page 3, added “*Study limitations include* *the inability of the biomarkers to distinguish different types of dairy foods and that most studies in the meta-analyses (including our novel cohort study) only assessed biomarkers at baseline, which may increase the risk of misclassification of exposure levels*.” |
| After the abstract, please add a new and accessible Author Summary section in non-identical prose. You may find it helpful to consult one or two recent research papers in PLOS Medicine to get a sense of the preferred style. | We have added a new Author Summary section per guidelines and recent research papers | Page 4, added Author Summary |
| Early in the Methods section of your main text, please state whether or not the study had a protocol or prespecified analysis plan, and if so attach the relevant document(s) as a supplementary file(s), referred to in the text. | We have now added the pre-specified analysis plan for the cohort study as supporting information (S1 Protocol).  We have specified early in the methods section of the systematic review that we had a protocol registered on PROSPERO. | Page 7 added “Analytic methods were pre-specified in a protocol (S1 Protocol).”  Added supporting information: S1 Protocol. Pre-specified analytical plan for 60YO cohort study |
| Please highlight analyses that were not prespecified. | Sensitivity analyses to adjust for self-reported dietary habits (vegetable, fruit and berries, lean fish, oily fish, and processed meat intake) was not prespecified. Adjustments for self-reported dietary habits did not alter the findings, as shown in Supplementary file 8. In addition, the test of differences in baseline characteristics across quartiles of 15:0 was not prespecified because it was requested by reviewer 2, and updated in Supplementary file 4. | Page 8, edited “We conducted sensitivity analyses by 1) adjusting for self-reported dietary habits… *(which was not prespecified)*…” |
| Are you able to update your search so as to include additional studies in the analysis? | We ran the search again up to 27 June 2021. We identified 388 search results but after screening, no new studies meeting the eligibility criteria. | Page 8, edited “A systematic literature search up to *27 June 2021* was conducted…”  Figure 3 flow chart of the systematic review was updated accordingly. |
| Throughout the text, please adapt reference call-outs to the following style: "... processing methods [4,5]." (noting the absence of spaces within the square brackets). | We have updated the referencing style as suggested | Changes made to the reference call outs in text (square brackets and an absence of spaces) |
| Please remove the information on funding, competing interests and data access from the end of the main text. In the event of publication, this information will appear in the article metadata, via entries in the submission form. Ethics information should be moved to the Methods section. | We have removed the information at the end of the main text as suggested. The revised information on data availability will be included in the article metadata during re-submission. Information on ethics is already in the methods. | We have deleted the sections on ethical approval, contributors, funding, competing interests, data sharing, contributors and dissemination to participants at the end of the main text on page 16 and 17. |
| Please review the reference list to ensure that all entries comply with journal style. Italics should be converted to plain text. Six author names should be listed rather than 3, followed by "et al.", where appropriate. | We have updated the reference list to ensure we comply with the journal’s reference style. | All references have been updated |
| Are references 17 & 48 missing journal names? | Added thanks. | Added journal names from ref 17 and 48 |
| Please use the journal name abbreviations "PLoS ONE" and "PLoS Med.". | Edited thanks | Edited journal names to abbreviations. |
| Please add completed STROBE (for the observational element of your study) and PRISMA (for the meta-analysis) checklists as supplementary files, labelled "S1_STROBE_Checklist" or similar and referred to as such in the Methods section. | Thank you, we have added the STROBE and PRISMA checklists as supporting information. We have also added the supporting information captions at the end of the manuscript. | Page 8, added “*This study is reported as per the Strengthening the Reporting of Observational Studies in Epidemiology (STROBE) guideline (S1 Checklist).”*  Page 8, edited “The systematic review followed the PRISMA guidelines *(S2 Checklist)*…” |
| In the checklists, please refer to individual items by section name, e.g., "Methods" and paragraph number, not by line or page numbers as these generally change in the event of publication. | Thank you for this suggestion. Added section number and paragraph number for STROBE and PRISMA checklists | Added: S1 Checklist STROBE statement for the reporting of cohort studies  S2 Checklist. PRISMA checklist |
| **Reviewer 1** | | |
| This article presents itself as two studies in one, which is not necessarily a bad thing (although may create potential for publication bias, if one of the research pieces is stronger that the other per se).  Did the authors consider splitting this into two separate papers for publication? | Thank you for pointing this out. While we did consider splitting our manuscript into two separate papers for publication, we felt it might be best to present the results from the 60YO cohort study in the context of previous studies in the field. By incorporating the novel results of the cohort study into a meta-analysis, we were able to provide the most comprehensive and up to date findings regarding the associations of dairy fat biomarkers and CVD risk and mortality. | No further changes. |
| Can the authors please comment on whether the 4150 included participants are representative of the wider population? | Given the high response rate (78%) and the random selection of the population-based cohort, the 4150 participants are likely representative of 60-year-olds in Stockholm County at the time of the baseline investigation. However, the generalizability to Swedes of other ages and in other geographical areas is less certain. | Page 14, edited text: “Firstly, the 60YO cohort is a large prospective study with a high participation rate *(78%),* which reduces the risk of *recall and* selection bias*es* and enhances the generalisability.” |
| Technically appropriate statistical models and methods have been applied by the authors. | Thank you. | No further changes. |
| "During follow-up, around 15% of participants died and a similar number of persons experienced a CVD event. Hence, we estimated the 15th percentile difference (PD) defined as the difference in time (months) by which 15% of exposed vs unexposed had died or experienced an incident CVD event. "  This is an interesting approach. Did the authors consider performing this analysis for a variety of percentiles and plotting these against time difference to visualise the trend of percentile differences? | Thank you for your suggestion. We did evaluate PD at percentiles lower than the 15^th^, which provided similar results. We have added a sentence to the methods section to acknowledge this. We did not attempt modelling PD at any greater percentiles as this would require extrapolating beyond the observed survival. | Page 7, added: “*Evaluation of PD at percentiles lower than the 15^th^ (i.e., 1^st^ to 14^th^) provided similar results*.” |
| "Three models were evaluated: 1) crude, without adjustments; 2) age- and sex adjusted; and 3) multivariable-adjusted including age, sex, BMI, smoking, physical activity, education, alcohol intake, diabetes, drug-treated hypertension and drug-treated hypercholesterolaemia as covariates".  This is a thorough and robust approach to take for the modelling. Did the authors consider including ethnicity or race as a covariate in the adjusted models? | Data on ethnicity or race were not available, but the majority (89%) of participants were either born in Sweden (81%) or Finland (8%). We have added a sentence to the limitations paragraph that the extrapolation to other ethnicities should be done with caution. We do agree that ethnicity/race is important to account for, and hence for the systematic review we conducted stratified meta analyses by geographical region. Still, as stated in the limitations, all studies included in the systematic review and meta-analysis were conducted in Western populations (i.e., USA, UK, or Scandinavia), which hampers generalizability to other populations and regions. | Page 17, added: “*The vast majority (89%) of the 60YO cohort were born in Sweden (81%) or Finland (8%) and extrapolation of the findings to other ethnic groups should be done with caution.*” |
| "Multiple imputations (n=20) were conducted to account for missing covariates."  Again, the authors have opted for a suitable and rigorous analytical approach here to account for missing data.  Did the authors complete any sensitivity analyses on this, perhaps by running the analysis with missing data excluded?  How much missing data was there? | We have added sentence about the degree of missing data. Due to very limited missing data, we decided it was not necessary to undertake a sensitivity analysis by excluding those with missing data. | Page 8, added “Less than 3% of the study population had missing values for ≥1 covariate, and the frequency of missing values in each covariate was <1%.” |
| "Serum 15:0 was assessed as a continuous variable (per interquintile range, IQR), defined as the range between the 90th and 10th percentiles) or a categorical variable (quartiles)."  The authors have demonstrated good practice by considering two ways of treating this variable within the modelling. Please note the typo of a surplus parenthesis. | Thank you. | We have removed the extra parenthesis on page 8 |
| "There was no violation of the proportional hazard assumption based on visual examination of Schoenfield residuals. Restricted cubic splines were used to evaluate potential nonlinear associations".  The authors have appropriately checked model assumptions for validity. | Thank you. | No further changes. |
| "We explored the associations between serum 15:0 and outcomes in subgroup analyses stratified by sex, BMI and serum n-3 PUFA subgroups (< median vs ≥ median)."  The subgroup analyses completed by the authors provide great insight to the data and patterns within. Did they additionally consider investigating age as a subgroup analysis? | Age was not included in the subgroup analyses as only men and women living in Stockholm that were 60 years old between 1 July 1997- 30 June 1998 were invited to participate in the study, hence our population had a very limited age range. We have clarified this detail in the methods now | Page 6, edited: “*One third of men and women aged 60 between 1 July 1997 and 30 June 1998 residing in Stockholm County (n=5,460) were* randomly selected *and* invited t*o participate in the study*.” |
| "We conducted sensitivity analyses by 1) adjusting for self-reported dietary habits (vegetable, fruit and berries, lean fish, oily fish, and processed meat intake), 2) excluding participants with prevalent CVD also from analyses of all-cause mortality (in line with analyses of CVD outcomes), 3) restricting analyses to the first 10 years of follow-up to minimise misclassifications attributable to exposure changes over time, and 4) excluding cases in the first two years of follow-up to avoid reverse causation because of undetected disease or presence of risk factors at baseline."  A thorough array of sensitivity analyses have been completed by the authors. | Thank you. | No further changes. |
| "The systematic review followed the PRISMA guidelines and was registered on PROSPERO 8 [CRD42020162551]. "  Can the authors please provide the PRISMA checklist and protocol within the supplementary material? | We have added the PRISMA checklist as well as the pre-specified analysis plan for the cohort study into the supplementary material. | Added the following supporting information:  S1 Checklist. STROBE statement for the reporting of cohort studies  S2 Checklist. PRISMA checklist  S1 Protocol. Pre-specified analytical plan for 60YO cohort study |
| "Two reviewers (SB and ZD) independently screened the studies for eligibility, extracted data, and assessed the quality of studies using the Newcastle-Ottawa Scale (NOS). "  Can the authors please clarify here how cases of disagreement, if any, were settled? Was this by consensus or by an independent third reviewer, perhaps? | We have added the appropriate text. | Page 9: added “*Disagreements were resolved by consensus or by involvement of a third reviewer (JW).*” |
| "Pooled associations of 15:0, 17:0, and t16:1n-7 with CVD outcomes and all-cause mortality were estimated using random effects meta-analysis."  The authors have applied a suitable modelling approach that can account for heterogeneity between the studies. | Thank you. | No further changes. |
| "Risk estimates (HR, odds ratio, or relative risk, RR) for each study were transformed to allow consistent comparisons between the top and bottom tertile of fatty acid distributions (Supplementary File 2)"  Can the authors please note in the limitations the risk of over/under stating these estimates by transforming them? | Thank you for this suggestion. The transformation of estimates assumes log-normal associations of fatty acid biomarkers with outcomes. This assumption appears reasonable since meta-analysing estimates per SD provided similar results to the quantile analysis. Nevertheless, we recognize the limitations of the transformation approach, especially in light of the non-linear association of serum 15:0 with all-cause mortality observed in 60YO. We have revised the limitation section to acknowledge limitations of the transformation approach. | Page 15-16, edited “Our study-level meta-analysis has known limitations *such as* potential for increased heterogeneity due to differences in study design (e.g. covariate selection) *and limited number of studies evaluating certain outcomes (e.g., all-cause mortality*). *We assumed log-linear associations of biomarkers with outcomes when transforming risk estimates to allow comparison of top vs bottom biomarker tertiles, which may over- or underestimate the estimates. However, meta-analysing risk estimates per biomarker SD in the subset of studies with relevant information available or retrieved from study authors provided similar results. Finally, the limited number of studies per lipid fraction, fatty acid, and outcome combination prevented us from evaluating non-linear associations using dose-response meta-regression. Many of the limitations to our meta-analysis could be addressed by de novo individual-level pooled analyses of prospective studies utilizing harmonised analysis protocols with pre-defined exposures, outcomes, and models*.” |
| "The I 2 and Q statistics were used to assess heterogeneity of included studies. Publication bias was assessed by visual inspection of funnel plots and statistically using Egger's and Begg's tests. Stratified meta-analyses were performed on subgroups defined by age, sex, follow-up duration, and geographic region. We repeated the meta-analysis using a fixed effects models in a sensitivity analysis."  The authors have demonstrated that they have followed a robust and rigorous analytical approach. | Thank you. | No further changes. |
| Furthermore, a thorough and informative selection of tables and figures have been presented within the manuscript. | Thank you. | No further changes. |
| **Reviewer 2- The study 'Biomarkers of dairy fat intake, incident cardiovascular disease, and all-cause mortality: a cohort study, systematic review, and meta-analysis' investigated the association of serum pentadecanoic acid (15:0), a biomarker of dairy fat intake, with incident CVD and all-cause mortality. The manuscript is well written, the analyses and conclusions are sound. The topic is of importance and of interest; it is commendable that the authors additionally present a meta-analysis in support of their findings.** | | |
| 1.Can the authors correlate serum cholesterol ester pentadecanoic acid (15:0) with a measure of diet quality (e.g. Healthy Eating Index, alternate Healthy Eating Index or DASH diet quality index) in the Stockholm cohort. | Thank you for your comments and suggestions.  The dietary data in the 60YO was collected using a crude questionnaire (17 questions) with limited information on serving sizes and thus did not allow quantitative estimation of food, nutrient or energy intake (Laguzzi et al., J Hum Nutr., 2016). Thus, we were unable to calculate diet quality scores. However, we did evaluate the intake frequency of certain food groups (i.e., vegetables; fruits and berries; fish; and processed meat) across quartiles of serum 15:0 (Supplementary file 4). We also evaluated the associations of dairy score based on intake frequencies (previously developed for 60YO) with serum 15:0 (Supplementary file 2). Importantly, adjusting for dietary habits did not have any substantial impact on the associations of serum 15:0 with incident CVD or all-cause mortality (Supplementary file 8). | No further changes. |
| 2.Sensitivity Analysis: Please additionally adjust your analysis for 'Total Energy Intake'. | As stated above (response to reviewer 2, comment 1), the dietary assessment tool utilized in 60YO prevented quantification of energy intake and thus we are unable to include this in our models. We did however include BMI and physical activity in statistical adjustments, both partly reflecting energy intake. Also, adjusting our main models for dietary habits (i.e., vegetables, fruits and berries, fish, and processed meats) provided similar results. | Page 15, added “*In future studies, a detailed dietary assessment should be conducted to investigate and adjust for potential interrelationship between intake of dairy fat, total energy, and macronutrients such as carbohydrates*.” |
| 3.Supplemental material: Please additionally explain how covariates were assessed in the Stockholm cohort. Also, please add a p-value to Suppemental Table 3 to assess if there were significant differences across quartiles. | We have added a supplementary file (1) detailing how covariates were assessed in the Stockholm cohort.  We have also added p-values in Supplementary file 4 (previously supplementary file 3). | Page 6, added “*The participants underwent a health screening, including blood sampling and completion of an extensive questionnaire (Supplementary file 1).”*  Page 7, edited “…3) multivariable-adjusted including age, sex, BMI….as covariates *(Supplementary file 1).*”  Added Supplementary file 1. |
| 4.If available, please add serum lipid level data (LDL, Tg, total Chol, HDL) according to quartiles of serum cholesterol ester pentadecanoic acid (15:0) to the results. | Thank you, we have added serum lipid levels (total cholesterol, HDL cholesterol, LDL cholesterol and triglycerides) into Supplementary file 4, as suggested. | Supplementary file 4 (previously supplementary file 3), added total cholesterol, HDL cholesterol, LDL cholesterol and triglycerides. |
| 5.If individuals consume more dairy fat (i.e. these individuals will have increased levels of serum cholesterol ester pentadecanoic acid (15:0)), they in turn will consume less amount of other macronutrients (in particular carbs). Can the authors show data to this respect ? Second, does carbohydrate quality modify serum levels of cholesterol ester pentadecanoic acid (15:0) ? | It is possible that a higher intake of dairy fat intake is associated with lower intake of specific macronutrients. As stated above (response to reviewer 2, comment 1), the dietary assessment tool utilized in 60YO prevented quantification of both the quantity and quality of carbohydrates. Thus we are unable to investigate relationships between carbohydrate intake and serum 15:0. However, intake frequencies of carbohydrate-rich foods like vegetables (including root vegetables), fruit and berries, and bread (“sandwiches”) were in fact higher in top vs bottom quartiles of serum 15:0 (Supplementary file 4), suggesting that dairy fat is not necessarily replacing carbohydrate in the diet of study participants.  While we were unable to adjust for dietary carbohydrate intake (quantity or quality) per se, our sensitivity analysis using models including dietary frequencies of fibre-rich foods provided results similar to our main analysis, and thus does not suggest that dietary fibre intake ( a reasonable marker of carbohydrate quality) is responsible for the observed inverse associations.  We have added new text highlighting the inability to conduct detailed dietary assessment analyses (due to the crude dietary assessment instrument) as a limitation of our study, and suggested that future investigation should focus on the potential interrelationship between intake of dairy fat and other macronutrients such as carbohydrates. | Page 15, added “*In future studies, a detailed dietary assessment should be conducted to investigate and adjust for potential interrelationship between intake of dairy fat, total energy, and macronutrients such as carbohydrates*.” |
| 6.Sensitivity analysis, please additionally adjust your analysis for 'carbohydrate intake'. | Please, see our response above (response to reviewer 2, comment 5). | No further changes |
| **Reviewer 3- The article "Biomarkers of dairy fat intake, incident cardiovascular disease, and all-cause mortality: a cohort study, systematic review, and meta-analyses" authored by Trieu et al. has been reviewed. Overall, this study provides additional evidence that markers of dairy fat intake seem to be associated with favorable cardiovascular outcomes and that there is no association between dairy intake and mortality. Indeed, the unusual but additional inclusion of the systematic review and meta-analyses supports the findings of the authors and strengthens the underlying message of the paper. However, I have some queries and suggestion below that are important to the manuscript. Good luck.** | | |
| Methods: Page 5 Line 10 - granted you have stated that measurement were only analyzed at baseline, but were follow-up plasma measurements taken? Was dairy intake and fatty acid composition assessed during follow-up to see if these measurements had changed much over time? | Thank you for your feedback and comments.  Dairy intake and serum 15:0 measurements were only taken at baseline. We have acknowledged that this is a study limitation in the discussion (on page 14). | No further changes. |
| Methods: Page 5 Line 15 - please detail the gas chromatography methods and isolation of serum cholesterol ester. | We have added some detail about the gas chromatography methods now. | Page 6, added “Briefly, serum cholesteryl esters were methylated, extracted in petroleum ether, evaporated under nitrogen, and then redissolved in hexane before analysis by gas chromatography using a 30-m glass capillary column coated with Thermo TR-FRAME; an Agilent Technologies system consisting of model GLAC 6890N, an autosampler 7683, and Agilent ChemStation; with a programmed temperature of between 150⁰C to 260⁰C.” |
| Why choose cholesterol esters. Phospholipids also contain 15:0 and 17:0. I assume it is partially because a blood sample is easier to get then other tissues. As a matter of interest, is s 15:0 and 17:0 measurable in RBCs? | Odd-chain fatty acids can be measured in adipose tissue, several circulating lipid fractions, including cholesterol esters, phospholipids, total plasma and also RBC [Imamura et al, doi: 10.1371/journal.pmed.1002670].Adipose tissue biopsies were not collected and fatty acid were measured in cholesterol esters, as has been done in other Swedish cohorts before (e.g., the [Uppsala Longitudinal Study of Adult Men](https://www.pubcare.uu.se/ulsam/)). We have previously observed strong correlations in Swedish populations between 15:0 in cholesterol esters and phospholipids [Marklund et al, doi: 10.3945/jn.117.254250], suggesting that the observed associations in 60YO would have been comparable if 15:0 had been assessed in phospholipids. | No further changes |
| What were exclusion criteria? Was BMI one of them, it seems unusual that the BMI range in table 1 is so low and that BMI > 33 was not reported in 60yo cohort. Indeed, obesity was also not reported in table 1. Certainly in 2020 over 54% of Swedish individuals over the age 60 have a higher BMI than 25 | We excluded participants without fatty acid data but we did not base any exclusion criteria on BMI. To further clarify the BMI distribution of our cohort - Table 1 reports the median BMI of 26.3 kg/m^2^, and the 10th and 90th percentile being 22.1 and 32.2 (i.e. 10% had BMI>32, and some did have BMI>33). This is similar to the current 2020 figure helpfully provided by the reviewer, as it suggests half of the sample had a BMI of 26.3 or more. The maximum BMI of the 4,150 participants included in our analysis was 51.8 kg/m^2^. | No further changes. |
| Move supplementary file 8 to the methods section | Thank you we have moved supplementary file 8 to figure 3 in methods section as suggested | Changed Supplementary file 8 to Figure 3. Flow chart of systematic review and selection process |
| Please provide figure legends for all figures. It is difficult to follow figures 1 and 2. | Apologies, these were accidentally removed. We have included figure legends at the bottom of the manuscript file on page 25. | Page 27, added “**Figure 1.** Hazard ratios of incident CVD as a function of serum pentadecanoic acid (15:0) in 60YO study. Data were fitted using Cox regression models adjusted for baseline age, sex, BMI, alcohol intake, smoking habits, physical activity, education, and prevalent hypertension, hyperlipidaemia, and type 2 diabetes. Dashed lines represent 95% confidence limits. The reference value of serum 15:0 is the 10th percentile (i.e., 0.17% of total fatty acids). The histogram shows the distribution of serum 15:0 in the cohort and the tick marks under the histogram indicate serum 15:0 levels of individuals who experienced an incident CVD event during follow-up.  **Figure 2.** Hazard ratios of all-cause mortality as a function of serum pentadecanoic acid (15:0) in 60YO study. Data were fitted using Cox regression models adjusted for baseline age, sex, BMI, alcohol intake, smoking habits, physical activity, education, and prevalent hypertension, hyperlipidaemia, type 2 diabetes, and CVD. Dashed lines represent 95% confidence limits. The reference value of serum 15:0 is the 10th percentile (i.e., 0.17% of total fatty acids). The histogram shows the distribution of serum 15:0 in the cohort and the tick marks under the histogram indicate serum 15:0 levels of individuals who died during follow-up.  **Figure 3.** Flow chart of systematic review and selection process.  **Figure 4.** Risk estimates for cardiovascular disease (CVD) incidence and all-cause mortality in the top tertile of pentadecanoic acid (15:0) relative to the bottom tertile. AT, adipose tissue; CE, cholesterol ester; CHD, coronary heart disease; CVD, cardiovascular disease; HF, heart failure; PP, plasma phospholipids; RBC, red blood cell (erythrocyte).  **Figure 5.** Risk estimates for cardiovascular disease (CVD) incidence and all-cause mortality in the top tertile of heptadecanoic acid (17:0) relative to the bottom tertile. AT, adipose tissue; CE, cholesterol ester; CHD, coronary heart disease; CVD, cardiovascular disease; HF, heart failure; PP, plasma phospholipids; RBC, red blood cell (erythrocyte).” |
| Introduction lines 2-14 - probably worth mentioning as an addition to the point made on line 9 that the evidence seems to suggest that fermented dairy products (yoghurt/cheese) in particular may be more protective than milk alone (<https://doi.org/10.3390/foods7030029>; <https://doi.org/10.1093/advances/nmz069>). | Thank you, we have incorporated the suggested references. | Page 5, added “*For example, fermented dairy products in particular, such as cheese and yoghurt, may be more protective than milk and butter.[8,9]*” |
| Editor comments:  Overall, I am supportive of the publication of this manuscript, but I have suggested revisions that require attention. | Thank you, we have made point-by-point response to reviewers’ suggestions now and revised the manuscript accordingly. |  |
