# Supplementary material for: Biomarkers of dairy fat intake, incident cardiovascular disease, and all-cause mortality: A cohort study, systematic review, and meta-analysis
Source: PLoS Med. 2021 Sep 21;18(9):e1003763. doi: 10.1371/journal.pmed.1003763 (PMC8454979; doi:10.1371/journal.pmed.1003763)
Supplement: Attachment — Submitted filename: Response to editors_final.docx [file pmed.1003763.s008.docx]

***Response to editors***

| **Editor comment** | **Authors’ response** | **Modifications to the manuscript** |
| --- | --- | --- |
| If available, please add a web address for the Karolinska Institute to your data statement | Thank you, we have now modified the data availability statement to include a web address for contacting the Karolinska Institute. | The following sentence about data availability will be included in the article meta data: “Data from the systematic review are within the manuscript and supporting Information files. Due to legal restrictions, some access restrictions apply to the remaining data underlying the findings. Requests could be sent to Karolinska Institutet, Contact details: Karolinska Institutet, 171 77 Stockholm, Sweden, Telephone: +46 8 524 800, <https://ki.se/en/about/how-can-we-help-you> ” |
| Rather than "60 year olds" in the abstract, please quote "... median age 60.5 years" or similar. | We have edited this now | Abstract, page 3, edited: “We measured 15:0 in serum cholesterol esters at baseline in 4150 Swedish *adults* (51% female, *median age 60.5 years*)” |
| Where you quote measures of risk in the abstract, please also quote p values as in the main text. | We have added this now. | Abstract, page 3, edited: “…(hazard ratio 0.75 per interquintile range; 95% confidence interval 0.61, 0.93, *P=0.009*)..” |
| Please adapt the wording in the "Conclusions" subsection of your abstract to note that you have also done a new cohort study. | Sure, we have added this now | Abstract, page 3, edited: “In a meta-analysis of 18 observational studies *including our new cohort study*…*”* |
| In the abstract and text you mention that "prospective" studies were included in the meta-analysis, and we ask you to remove this word from the abstract. Although we are aware that views differ, judging from the description of the research designs included in the main text we doubt that all these studies meet the definition of a prospective study that we generally use at PLOS Medicine. | Sure, we have removed “prospective” from the abstract as suggested and specified study types included. | Abstract, page 3, edited to remove the term prospective and specified study types:  Line 14: “In meta-analyses including our Swedish cohort and 17 cohort, case-cohort, or nested case-control studies,”  Line 23: “In a meta-analysis of 18 observational studies…” |
| In the author summary, we suggest "it is important ..." rather than "it is critical ...". | We have edited this now. | Author summary, page 4, edited: “… it is *important* to advance our understanding of the impact of dairy fat on CVD risk.” |
| Please adapt the reference call-outs throughout the ms to the following style (noting the absence of spaces within the square brackets): " ... [4,6,7]. For example ...". | Thanks, we have updated the references to this format with no spaces within the square brackets. | Updated references in text |
| In the reference list, please remove the information on competing interests from references 3 & 61, and any other relevant citations. | We have removed competing interests from reference 3 and 61 no, reviewed the reference list. | Revised reference list as suggested |
